# Acupuncture Points of the Horse’s Distal Thoracic Limb: A Neuroanatomic Approach to the Transposition of Traditional Points

**DOI:** 10.3390/ani2030455

**Published:** 2012-09-17

**Authors:** Lisa S. Lancaster, Robert M. Bowker

**Affiliations:** 1 Medical Acupuncture for Veterinarians Course, Colorado State University, Fort Collins, CO 80525, USA; 2 Department of Pathobiology and Diagnostic Investigation, College of Veterinary Medicine, Michigan State University, G205, East Lansing, MI 48824, USA; E-Mail: Bowker@cvm.msu.edu

**Keywords:** equine acupuncture, equine laminitis, equine neuroanatomy, veterinary medical acupuncture

## Abstract

**Simple Summary:** Anatomy of the equine foot is not precisely analogous to anatomy of the human hand and foot. Thus acupuncture points transposed from human acupuncture maps may not be functionally similar in the equine digit. Veterinarians strive to select points based on what is currently known about the anatomy and physiology of the equine foot, despite the lack of research evidence to use as guidance. This paper discusses the anatomy and physiology of the horse’s foot and presents a neuroanatomically based modification of some traditional point locations including several newly proposed points. The paper also presents neuroanatomically based clinical suggestions for laminitis treatment.

**Abstract:** Veterinary acupuncture charts were developed based on the concept of transpositional points whereby human acupuncture maps were adapted to animal anatomy. Transpositional acupuncture points have traditionally been placed in specific locations around the horse’s coronet and distal limb believed to be the closest approximation to the human distal limb points. Because the horse has a single digit and lacks several structures analogous to the human hand and foot, precisely transposing all of the human digital points is not anatomically possible. To date there is no published research on the effect of acupuncture treatment of the equine distal limb points. This paper presents a modified approach to equine distal limb point selection based on what is known from research on other species about the neuroanatomic method of acupuncture. A rationale is presented for modification of traditional equine ting points as well as additional points around the hoof and distal limb that do not appear in the standard textbooks of equine acupuncture. The anatomy and physiology of the equine foot likely to be affected by acupuncture are briefly reviewed. Modified neuroanatomic points are proposed that may be more accurate as transpositional points. As an example of clinical application, a neuroanatomic approach to acupuncture treatment of equine laminitis is presented.

## 1. Introduction

Acupuncture has been used on animals for thousands of years but only since the 1970s in the United States have there been professional organizations dedicated to training veterinarians in acupuncture theory and practice [1]. Acupuncture charts were developed based on the concept of transpositional points whereby human acupuncture maps were adapted to animal anatomy. Transpositional acupuncture points have traditionally been placed in specific locations around the horse’s foot and distal limb believed to be the closest approximation to the human hand and foot points. Because the horse has a single digit and lacks structures analogous to the human hand and foot, points traditionally placed distal to the equine carpal and tarsal joints may not have the same physiologic effects as points on the human wrist, hand, ankle and foot.

This paper begins with a description of the main anatomic structures within the foot that are likely to be affected by acupuncture needles placed proximal to the hoof capsule. Then a rationale is presented for using traditional points as well as modified placement and newly proposed points. Because there is limited research on equine acupuncture, discussion of acupuncture mechanisms of action for the equine foot is based on known physiological effects in other species. The final section of this paper presents clinical suggestions for point selection in treating equine laminitis. 

## 2. Equine Foot Anatomy and Physiology

**Figure 1:**
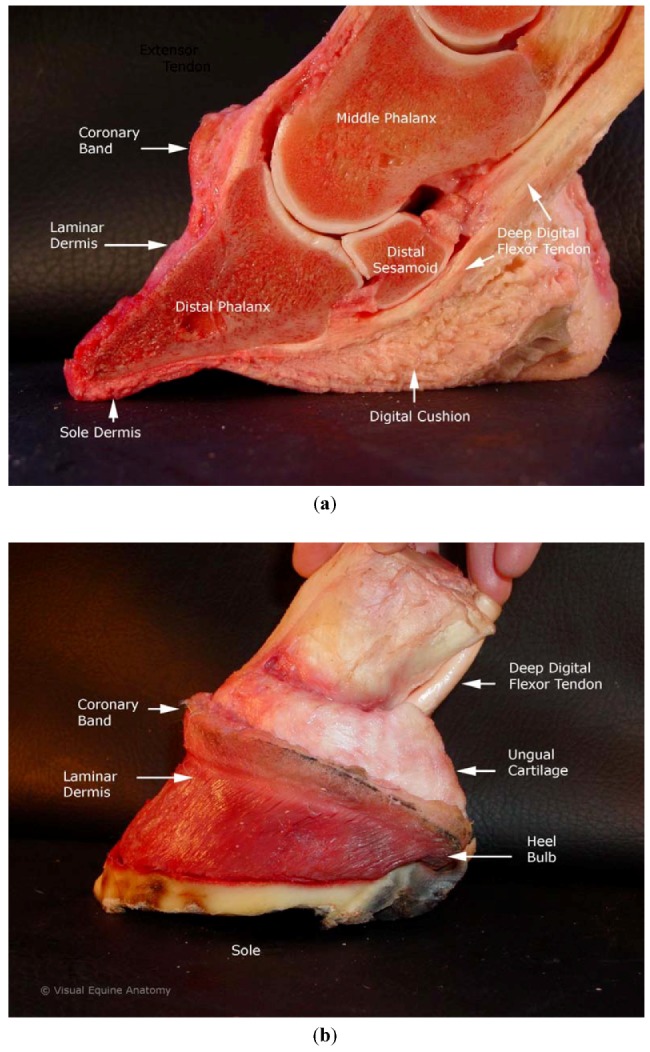
(**a**) Equine foot specimen sagittal section with hoof capsule removed. Labels identify structures likely to be influenced by acupuncture needles in coronet and nerve block point locations. (**b**) Equine foot specimen with hoof capsule and skin removed, laminar and coronary dermis left intact.

The equine digit refers to the structures distal to metacarpophalangeal or metatarsophalangeal joint (fetlock) (Figure 1(a,b)). The equine digit includes four bones: the first, second and third phalanges, and the navicular bone (distal sesamoid). The term foot refers to all structures enclosed within the hoof capsule. The hoof capsule and its internal contents include the distal phalanx and distal sesamoid bone, and dermal and epidermal components of the coronary region, hoof wall, frog, bars, sole, white line, digital cushion, ungual cartilage, and laminae. The region where the proximal hoof wall meets the haired skin is called the coronet or coronary band. The digit has ligaments between bones as well as between bone and ungual (commonly termed lateral) cartilage. There are no muscles distal to the carpus or tarsus. The tendons within the foot have their muscle bellies on the proximal limb. 

### 2.1. Vasculature

Blood supply to the thoracic limb comes from the axillary artery, which becomes the median artery that passes through the carpal canal. Proximal to the fetlock the median artery branches into medial and lateral palmar digital arteries. The digital arteries circle around the distal phalanx in a coronary and distal margin circumflex formation. Multiple branches form anastamotic networks throughout the foot.

There are more veins than arteries in the foot and all vessels are highly sensitive to vasoactive substances. There are three divisions of the valveless venous plexus system [2]: (a) palmar venous plexus in the sole dermis and axial edges of the ungual cartilages, (b) coronary venous plexus around coronary band and abaxial collateral cartilages (this is the one we can most directly access with an acupuncture needle), and (c) the dorsal venous plexus in the deep laminar dermis. The laminar and coronary dermis contain arteriovenous anastamoses (AVAs) that function as part of the pressure regulating mechanism and thermoregulation within the foot [3].

### 2.2. Innervation

Distal thoracic limb innervation is primarily from the median nerve, which is the largest branch of the brachial plexus, arising from segments C8-T2. There is also some contribution from the ulnar nerve, arising from segments T1-2. The radial nerve in the equine does not travel distal to the carpus. The median nerve splits into medial and lateral palmar nerves distal to the carpus, and it is termed the palmar digital nerve (PDN) distal to the MCP joint. Branches of the ulnar nerve also contribute to digital innervation. The metacarpal nerves do not always reach the foot.

Branches of the PDN are clinically important for diagnostic analgesia. Dorsal branches of the PDN form just proximal to the fetlock (MCP) joint. Medial and lateral branches are not mirror images. The PDN on the medial side is a larger nerve and has more branches extending into the hoof capsule. The dorsal branches of the PDN are also different from side to side. The lateral dorsal branches terminate at mid-pastern level proximal to the hoof capsule. The medial dorsal branches of the PDN extend into the foot but the lateral branches do not. These medial and lateral differences were documented in the 1970s based on gross anatomic studies using a dissection scope [4]. There is more recent histologic evidence that many smaller branches innervate more structures within the foot than previously recognized [5].

### 2.3. Nerve Fiber Types and Neuropeptides

The palmar digital nerves contain sensory and autonomic fibers. The unmyelinated nerves contain approximately 75% sensory and 25% sympathetic fibers. Digital vessels, including the AVAs are innervated by autonomic vasomotor nerves [3]. Unmyelinated nerves convey various sensations to the spinal cord including nociception and high-threshold mechanoreception. The myelinated fibers are less numerous and convey information more rapidly to the spinal cord. Research has identified several neuropeptides in the equine foot, the most studied include neurokinin A, calcitonin gene related peptide (CGRP) and substance P, all of which have been identified within the palmar digital nerves [6]. These neuropeptides in the sensory nerves are potent vasodilators. Vasoconstriction is mainly sympathetically mediated by adrenergic nerve fibers.

### 2.4. Mechanoreceptors

The equine foot has no muscles and therefore no motor nerves but it is still an important sensory organ. The outer hoof wall and sole that come in contact with the ground are not reactive to touch or noxious stimuli (hence we can nail shoes to the bottom of the foot and attach plates or drill holes in the outer wall without causing pain to the horse). Proprioceptors and other mechanoreceptors and nociceptors have been identified inside the foot [6]. Mechanoreceptors respond to touch, pressure, vibration or other physical deformation of the hoof capsule and its contents, allowing the horse to adjust stance and foot placement according to varying environmental conditions.

Mechanoreceptor types identified within the horse’s foot include Pacinian and Ruffini corpuscles [5]. Pacinian corpuscles are rapidly adapting receptors that respond to abrupt pressure changes and vibration during movement as the foot repeatedly touches and leaves the ground. Pacinian corpuscles are supplied by large diameter myelinated nerves and are located in the caudal frog, heel bulbs, axial to the lateral cartilages, and around the secondary tendon of the deep digital flexor tendon (DDFT) just proximal to the navicular bone.

Ruffini corpuscles respond to different stimuli than Pacinian corpuscles do. The Ruffini corpuscles respond to sustained pressures when the horse is standing still. These receptors have been identified within neurovascular bundles and the solar dermis.

## 3. Modified and Newly Proposed Acupuncture Points

Transpositional points for the equine digit have traditionally been placed around the coronet region despite the markedly different anatomy of the equine from the human digit [7]. The neurovascular anatomy of the pelvic and thoracic equine limbs is similar distal to the tarsus and carpus. This paper limits the discussion to points on the thoracic limb but similar principles should apply to the pelvic limb. There are six ting points on the human hand (and 6 on the foot). The ting points are located on medial or lateral sides of the digits at the skin-nail junction at the proximal extent of the nailbed. The neurovascular bundle is the relevant anatomy underlying these points. It is not known if transposed points have the effects in the single-toed animals as they have in other species.

### 3.1. Transpositional Ting Points and Coronet Points

Because the horse has a single digit, all 6 ting points have traditionally been placed around the coronary band region as a way to accomplish transposing all the human ting points despite the lack of analogous anatomic structures. As in the human digits, in the equine single digit a neurovascular bundle courses along the medial and lateral sides. Therefore it may be more neuroanatomically accurate to say that the horse has only two transpositional ting points on each limb. These newly proposed transpositional ting points would be where a distal palmar digital nerve block is placed [8]. Coronet points (previously transpositional ting points), although not analogous to any points on the human digit, are still valid acupuncture points given the importance of the venous plexus structures underlying the coronet. 

**Figure 2:**
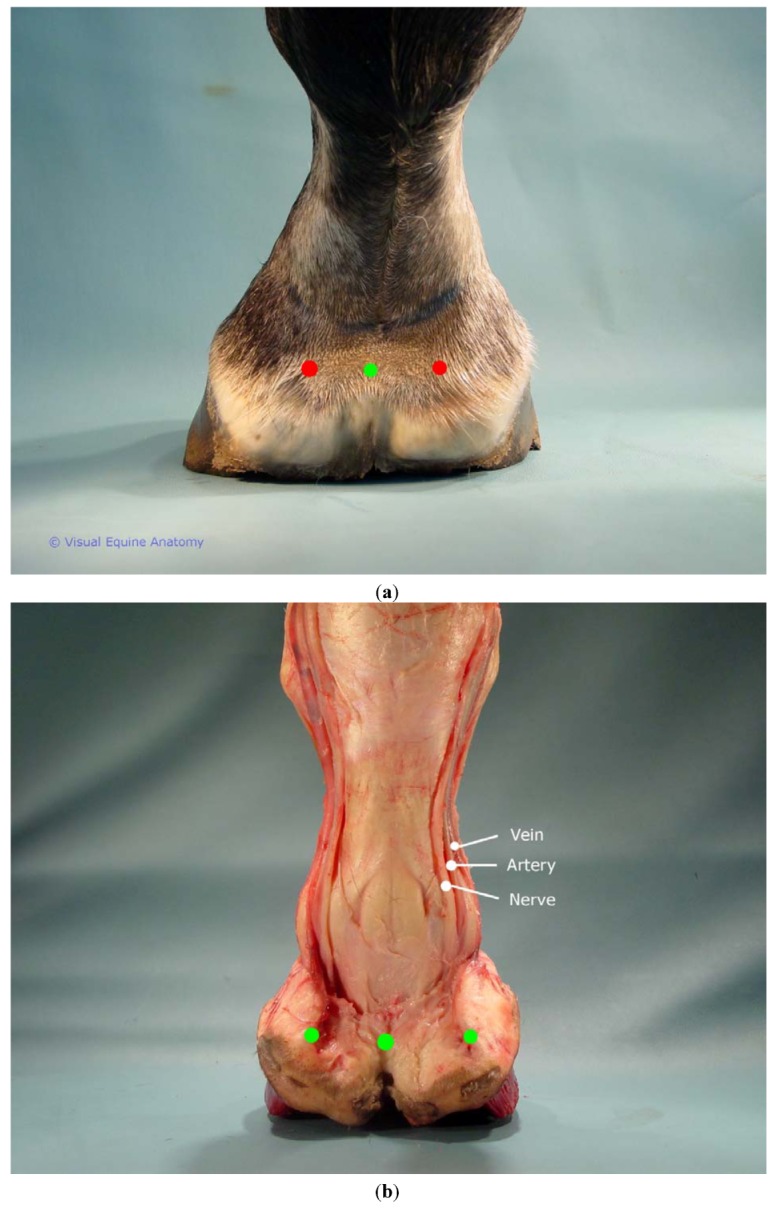
(**a**) Equine palmar foot coronet points shown on cadaver intact foot. (**b**) Dissected specimen showing placement in relation to ungual cartilage and heel bulb.

**Figure 3:**
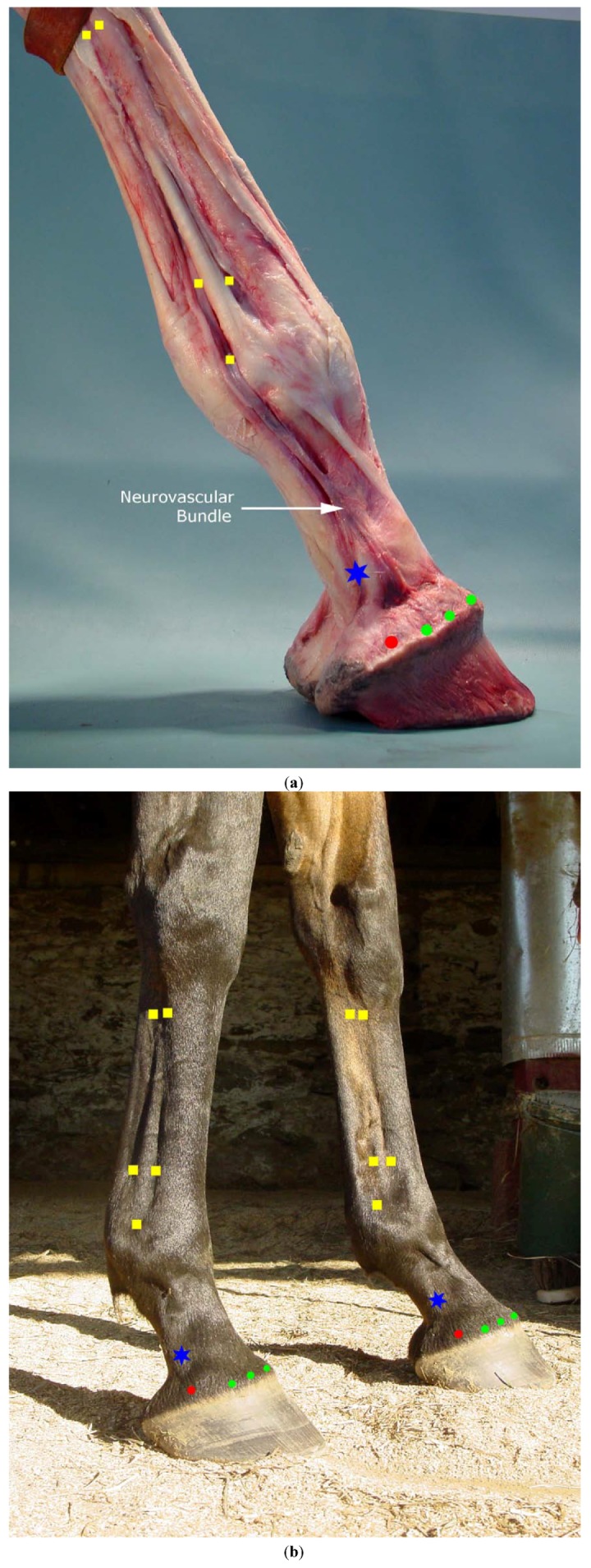
(**a**) Equine foot and lower limb specimen distal to carpus with epidermal tissues removed. (**b**) Live horse medial and lateral views showing location of proposed acupuncture points. Green dots: previously termed ting points, now coronet points. Red dots: additional coronet point over ungual cartilage. Blue star: equine ting point over PDN nerve block location. Yellow squares: additional block points, distal point—abaxial block, proximal to MCP joint—low 4 point block, distal to carpus, high 4 point block.

The venous plexus most directly accessible to acupuncture treatment is the coronary venous plexus around the circumference of the foot. On palpation the tissue just proximal to the hairline on the dorsum of the foot has a soft tissue feel but the plexuses extend around the entire foot over the quarters and heel bulbs where the proximal portion of the collateral cartilages gives this plexus a harder, less distinct tissue feel. Traditionally ting points were placed at the dorsal and palmar midline, and at medial and lateral locations. The precise site varies in different texts but usually the caudal medial and lateral points are placed cranial to the lateral cartilages [7]. Distinctive tissue feel (described in medical terms as edematous or atrophied) was an important diagnostic marker for traditional Chinese medicine [9]. The cartilaginous hard tissue feel of the horse’s palmar coronet region lacks such distinctive tissue feel which may be a reason why these equine points were not over the ungual cartilage. Proposed coronet points include all points around the circumference of the foot that includes previously termed ting points and heel bulb points, plus the addition of points over the ungual cartilage which is an area rich in superficial innervation (Figure 2(a,b) and Figure 3(a,b)).

### 3.2. Proposed Additional New Points: Nerve Block Points

In re-evaluating the transpositional ting points for horses it may be anatomically accurate to designate only two points per limb as true ting points, placing those at the location for the PDN block. Additional newly proposed points would not be called ting points because they are not neuroanatomically analogous to the human ting points. However the palmar nerves and palmar digital nerves, clinically important in equine lameness, can be stimulated with acupuncture needles at sites where nerve blocks are placed. For example blocks between the MCP joint and the carpus are conventionally termed abaxial, low palmar (low 4-point), high palmar (high 4 point), and lateral palmar nerve blocks [8]. The block points are located over main superficial nerve trunks. Discrepancies over acupuncture point names can be a source of confusion [10]. To facilitate efforts at standardization of acupuncture points it may help to chose point names that clarify the anatomic location which in turn assists the practitioner in devising a rational point selection protocol.

Points on the medial and lateral sides of the foot can be used. However anatomic studies on equine thoracic limbs have shown that the medial PDN is larger, has more branches, and innervates more structures inside the foot [4,5]. Although research is needed to identify what, if any effect results from acupuncture needles placed in this region, it is likely that if there is an effect, it may be increased when treating the medial side where more nerve branches have been identified.

#### 3.2.1. Proposed Modification to Traditional Points Large Intestine 4 (LI4) and Small Intestine 3 (SI3)

In humans, the acupuncture point LI4 is on the hand in the first interosseous muscle (the muscle between the thumb and index finger). This is a commonly used acupuncture point for diverse physical and mental disorders and has been shown in imaging studies to influence specific brain regions [11,12]. Research on acupuncture mechanisms of action has shown that certain tissue characteristics at acupuncture points account for the varied physiological effects [13]. Although animal models (mostly rodent) as well as human models have been used in research, rodents, despite their anatomical differences from humans are still more anatomically similar to humans than horses are. The anatomical differences are particularly distinct in the hand and digits. One of the tissue features believed to be important in the mechanism of action for the human point LI4 is the motor innervation [14]. The significant physiological effects of LI4 in humans are a result of this point having several specific features that are not present in the equine distal limb. The first interroseus muscle in the human hand is a major motor point. A needle placed into this muscle mass will interact with muscle spindle afferents inciting local, segmental, and suprasegmental responses. The first interosseous muscle in the human hand is innervated by the radial, ulnar and median nerve.

In horses there is no 1st digit, no effective muscle tissue distal to the carpus and the radial nerve does not extend distal to the carpus. Because the horse has no thumb or index finger, the transpositional point was placed just distal to the proximal extent of the second metacarpal bone in the groove between the second and third metacarpal bones.

Lacking significant interosseous muscle mass into which a needle could interact with muscle spindle afferents, it is not clear how much widespread effect LI4 would have on a horse. There are superficial nerves in this region where LI4 is traditionally placed in the horse. It is likely to have local tissue effect as would any cutaneous input. But the diverse effects of this point in humans are unlikely to be found in horses. Research on LI4 in humans suggests that its dense innervation explains the segmental and suprasegmental effects that result from significant somatic afferent stimulation [11,13].

Large Intestine 4 in the horse in its current transpositional location is likely to have local effects on the small nerves and vessels in the metacarpal region. The palmar nerve at this location does not extend distally into the foot therefore a modified location for this point that might have more impact on the foot would be to make this a 4 point acupuncture point corresponding to the high 4 point (high palmar) nerve block. The 4 points would include the original medial location just caudodistal to the head of the 2nd metacarpal bone. The transpositional point was placed on the medial side of the horse’s limb because that is analogous to the radial aspect of the human hand. Given that the horse’s digit is symmetrical, the point could logically be placed bilaterally to potentially enhance the neurovascular input. The other three points would include the lateral side caudodistal to the 4th metacarpal bone and medial and lateral points at the same level caudal to the interosseous suspensory ligament (in the depression where the main trunk of the palmar nerve and vessels course that supply the foot) (Figure 3(a,b)).

#### 3.2.2. Small Intestine 3 (SI3)

In humans the acupuncture point Small Intestine 3 (SI3) is located proximal to the fifth metacarpophalangeal joint on the ulnar aspect of the hand. Because the horse does not have a 5th metacarpal bone, the transpositional point on the horse was placed distal to the distal aspect of the fourth metacarpal bone in the depression between the third metacarpal bone and the suspensory ligament. The transpositional point was placed on the lateral side of the equine limb to correspond to the ulnar aspect of the human hand. As noted with LI4, there are no muscle spindle afferents to be stimulated in equine points distal to the carpus, therefore less afferent information may be transmitted to segmental and suprasegmental areas in the equine central nervous system. The point should still have value locally to modulate distal limb neurovasculature. As mentioned for LI4, since the limb is symmetrical, SI3 could be placed bilaterally. It is possible that the medial side would be even more influential for the foot neuroanatomically since there are more branches of the PDN on the medial side. Additionally, as with LI4, it is also possible that the point might be more influential if placed palmar to the suspensory ligament (in the groove between the suspensory ligament and the digital flexor tendons) because this is the location of the larger nerves and vessels supplying the foot. Therefore the proposed modification for SI3 is to make this a 4 point acupuncture point in the location of the low 4 point (low palmar) nerve block (Figure 3(a,b)).

### 3.3. Possible Neurophysiology of Newly Proposed Points

In speculating on the neurophysiology of these newly proposed points over superficial peripheral nerves, consider acupuncture points on the face. Many of the face points are precisely where dental and ophthalmic nerve blocks are placed. Research on canine transpositional point periorbital anatomy has shown that some modification is needed to assure needle placement over specific anatomic structures [15]. Although no such anatomic research has yet been conducted on any equine points, because acupuncture is known to influence neurovascular physiology [16] it is logical to place needles over neurovascular structures. 

It is likely that limb points would be effective where peripheral nerve blocks are placed for diagnostic analgesia. Electrophysiologic study of the equine thoracic limb identified autonomous zones—regions of cutaneous innervation supplied by a single nerve—for the ulnar and median nerves that arise segmentally from C8-T2 [17]. Therefore acupuncture needles placed in these cutaneous areas should have local, segmental and possibly suprasegmental effects via influences on these brachial plexus nerves. Segmental effects are likely to include autonomic effects in the feet and motor effects in the proximal limb muscles. 

Equine digital blood flow has been the subject of much research. Controversy persists about whether vasoconstriction [18] or vasodilation [19] is a prodromal event in laminitis. Alterations in blood flow exist at the level of the microvasculature and the digital vessels with some research showing a predisposition for venoconstriction in the microvasculature of the laminae [20]. Knowing precise details of vascular dysregulation may not be essential to choose acupuncture points for equine foot disease. Future research will test this in the horse but because a general principle of acupuncture effects includes normalizing vasomotor physiology, it would be expected to facilitate vascular homeostasis in the equine foot.

Research on rats has shown that acupuncture affects regional blood flow to brain, viscera and skeletal muscle via segmental and suprasegmental autonomic reflexes [21]. It is likely that these same reflex mechanisms in horses would respond to acupuncture needles with similar reactions. However the lack of muscle and therefore motor nerves in the distal limb may result in different effects than are seen in human hand and finger points.

Myofascial trigger points—discrete local areas of abnormal muscle fibers—have been described in horses [22] and would therefore presumably be amenable to acupuncture treatment. It is not always possible to needle animals deeply directly into trigger points as they may be less tolerant than human patients are of painful treatments. However it does seem clinically possible to reduce palpable trigger points in horses even with superficial needling. This may be explained in part by a proposed mechanism of acupuncture involving mechanoreceptor activation via needle grasp into subcutaneous tissue at fascial planes [23,24].

Research on acupuncture analgesia has identified complex processes of somatic afferent impulses integrating at different levels of the central nervous system to modulate pain in ascending and descending pathways [25]. Pain control is one of the most researched effects of acupuncture and thus promising as part of multimodal treatment for equine laminitis. 

Research on equine acupuncture has investigated its use for gastrointestinal [26], pulmonary [27] and reproductive conditions [28]. Some equine pain research has been done on experimental visceral pain [29], experimental lameness [30,31] and on naturally occurring back pain [32,33]. However there is not yet published investigation of how distal limb acupuncture points might work to influence foot physiology and no research on the use of acupuncture for laminitis. 

## 4. Clinical Suggestions for Laminitis Treatment

Laminitis is a disease that involves loss of integrity of the laminar junction. The laminar tissue loses its connection between the hoof wall and the distal phalanx resulting in pain and lameness [34]. There are multiple causes [35], including metabolic [36,37] and mechanical [38], that can result in failure of the laminar bond. Laminitis is an important problem in the equine industry and in the past 40 years there has been considerable research effort on this disease [39]. Because horses cannot sit down, or readily lie down like dogs can do, a horse with severe laminitis has limited postural options for relieving itself of pain. There is no precisely analogous human disease, although Raynaud’s syndrome has been compared to equine laminitis [40]. But a human with pain in the fingers or toes does not have to support their entire weight on those painful fingers or toes. A laminitic horse can experience extreme foot pain and may be euthanized if the pain becomes unresponsive to treatment. Therefore pain control is the primary goal of any laminitis treatment. Multi-modal treatment is always indicated although there are toxicity concerns with some of the pharmaceutical options available for treating musculoskeletal pain [41]. 

Laminitis is a treatment challenge because of its multisystem involvement and the complex nature of laminitic pain that includes inflammatory, nociceptive and neuropathic pain [42,43,44]. But this is what makes it a particularly appropriate disease for acupuncture as it is known to influence multiple body systems and modulate complex pain pathways [21].

Acupuncture treatment is based on point selection for local, segmental, and suprasegmental influences. In selecting points most likely to be effective, we also consider autonomic influences as well as whole body myofascial dysfunction. 

For example, the laminitic foot itself might be most directly influenced by needling the neurovascular region just proximal to the foot (coronet and PDN points), as well as along the entire neurovascular channel up the limb (additional block points). Thinking about this laminitic foot segmentally, assuming it is a thoracic limb which is most likely to be affected with laminitis, caudal cervical and shoulder points might be selected. For suprasegmental influence we might choose homeostatic points that target systemic inflammation and GI issues which can be comorbid conditions in laminitic patients. We can consider treating overall myofascial tension as another way to influence autonomic tone. Needling paravertebral points (bladder line) can decrease dorsal horn windup that is occurring as a direct result of foot pain. Paravertebral muscles may also be part of myofascial pain syndromes in laminitic horses, and thus respond directly to trigger point release, which in turn reduces sympathetic tone. 

If the laminitis is in one foot only, if not resolved quickly, the contralateral foot is at risk for support limb laminitis. Treating bilaterally is wise even if clinical signs are only in one limb. Treating digital flexor muscles can help some horses. Because the foot lacks muscle, there are limited responses of the foot itself to pain within the hoof capsule. Clinically there appears to be a reflexive shortening of the digital flexor muscles although it is not effective in unloading the painful parts of the foot. Considering the state of excess tone, the physiology of the muscle is likely to be in an energy deprived state found in trigger points in other species. These horses can be treated with acupuncture needles in the antebrachium. Textbook elbow points as well myofascial trigger points primarily in the muscle bellies of the digital flexors can be treated. 

In addition to contracting muscles of the digital flexors, another response to foot pain is for the horse to alter its overall posture. It may lean back, in the classic severe founder stance, or it may make subtle changes to the neck and back that result in trigger point pain that can persist even as the foot begins to heal. In chronic cases there can be more impressive clinical results from treating myofascial trigger points than treating distal limb points. Even though in severe acute cases there may be altered stance, if it hasn’t been that way for long, there may not be significant myofascial problems throughout the body.

Mechanoreceptors in the foot are affected by how the horse stands and moves [5]. So while we can’t needle them directly, or even necessarily affect these tissue receptors by needling sensory nerves that supply them, consider how we can indirectly influence mechanoreceptor activation by changing how the horse loads the feet. If acupuncture can relieve pain and deactivate trigger points, the horse has a chance to normalize posture. It is more important for a laminitic horse to stand comfortably than to walk without pain. Of course ideally we want horses to walk without pain. But in many cases this is not a realistic clinical goal. Yet it is unacceptable to leave horse in pain even when it is not moving. Most domestic horses, whether or not they are in pain, stand around more than they move. The effect of posture on digital physiology can therefore be more important than what happens when they move.

To sum up, the primary goal in treating laminitis is pain control. Acupuncture’s well documented effects on pain control in other species, as well as in horses (although not tested for foot pain) suggests this may be the most obvious benefit of acupuncture for laminitis. Once pain is controlled, other clinical goals may be amenable to acupuncture including treatment of comorbid conditions and normalizing foot physiology. Achieving these clinical goals may be facilitated when treatment is focused on the neuroanatomical location of acupuncture points. This approach will permit the clinician to customize treatment of a complex disease such as laminitis by choosing acupuncture points to stimulate key neurovascular, muscular or visceral structures.

## 5. Conclusions

This paper reviewed anatomy of the equine foot and limb in order to understand neuroanatomic rationale for needle placement. Recognizing that we lack research on equine acupuncture, it is important to think carefully in selecting reasonable locations for needle placement. This paper suggested that the neuroanatomic transpositional ting points in the horse are bilateral points located proximal to the ungual cartilage where a PDN block would be placed. Additional nerve block points can be needled at or near the block sites including abaxial, low palmar and high palmar (low and high 4 point nerve blocks). Modification of point placement and point naming for previously termed transpositional points LI4 and SI3 may be clinically helpful. Naming of these points in anatomic terms designated for diagnostic anesthesia will facilitate ease of use because the location and potential clinical uses are implied by the names high palmar (LI4) and low palmar (SI3). Modification includes using those points bilaterally and considering needle placement both dorsal and palmar to the suspensory ligament that will provide neural input to metacarpal as well as digital nerves. Venous plexus points around the coronet as well as over the ungual cartilages are also appropriate for foot treatments. Also important in laminitis are myofascial trigger points, viscerosomatic, and homeostatic points to treat components of or sequelae to foot disease. Future research may answer questions about which point or points are most influential for modulating equine foot physiology thus aiding clinicians in formulating evidence based acupuncture treatment protocols.
